# Hypnotherapy self-exercises with audio files for children and adolescents with disorders of gut-brain interaction—a study of feasibility and preliminary effects in Sweden

**DOI:** 10.1177/17562848251381141

**Published:** 2025-09-28

**Authors:** Tea Soini, Frida Andersson, Maria Lalouni, Marianne Bonnert, Siri Voghera, Brjánn Ljótsson, Marc A. Benninga, Arine M. Vlieger, Helena J. Rolandsdotter, Agneta Uusijärvi, Ola Olén

**Affiliations:** Division of Clinical Epidemiology, Department of Medicine, Solna, Karolinska Institutet, Maria Aspmans gata 16, Stockholm 171 77, Sweden; Department of Clinical Science and Education, Södersjukhuset, Karolinska Institutet, Stockholm, Sweden; Division of Clinical Epidemiology, Department of Medicine, Solna, Karolinska Institutet, Stockholm, Sweden; Pediatric Gastroenterology and Nutrition Unit, Sachs’ Children’s Hospital, Stockholm, Sweden; Department of Clinical Neuroscience, Division of Neuro, Karolinska Institutet, Stockholm, Sweden; Department of Clinical Neuroscience, Centre for Psychiatry Research, Karolinska Institutet & Stockholm Health Care Services, Stockholm, Sweden; Division of Clinical Epidemiology, Department of Medicine, Solna, Karolinska Institutet, Stockholm, Sweden; Department of Clinical Neuroscience, Division of Psychology, Karolinska Institutet, Stockholm, Sweden; Pediatric Gastroenterology and Nutrition, Emma Children’s Hospital, Amsterdam University Medical Center, University of Amsterdam, Amsterdam, The Netherlands; Department of Pediatrics, St. Antonius Hospital, Nieuwegein, The Netherlands; Department of Clinical Science and Education, Södersjukhuset, Karolinska Institutet, Stockholm, Sweden; Pediatric Gastroenterology and Nutrition Unit, Sachs’ Children’s Hospital, Stockholm, Sweden; Astrid Lindgren Children’s Hospital, Karolinska University Hospital, Huddinge, Sweden; Division of Clinical Epidemiology, Department of Medicine, Solna, Karolinska Institutet, Stockholm, Sweden; Pediatric Gastroenterology and Nutrition Unit, Sachs’ Children’s Hospital, Stockholm, Sweden

**Keywords:** disorders of gut-brain interaction, feasibility study, hypnotherapy

## Abstract

**Background:**

Gut-directed hypnotherapy is effective for treating children with disorders of gut-brain interaction, but is currently unavailable in Sweden.

**Objectives:**

To evaluate the within-group effect and feasibility of a Swedish adaptation of an audio-based gut-directed hypnotherapy program.

**Design:**

Uncontrolled within-group feasibility study.

**Methods:**

Children and adolescents (aged 8–17) diagnosed with irritable bowel syndrome, functional abdominal pain, or functional dyspepsia (per Rome IV criteria) participated in a 12-week online hypnotherapy program. The program used audio files that were translated and adapted from a validated Dutch protocol. Data were collected at baseline, during treatment, and post-treatment. The primary outcome was gastrointestinal symptoms, measured by the PedsQL Gastrointestinal Symptoms Short Scale. Secondary outcomes included pain intensity and frequency, quality of life, stress, depression, anxiety, school absenteeism, and treatment credibility and satisfaction. Analyses with linear mixed models were used to estimate means, standard deviations, and effect sizes (Cohen’s *d*).

**Results:**

Of the 32 patients included in the study, 25 (78%) completed the program and provided post-treatment data. Significant improvements in PedsQL gastro score were observed, with moderate effect size in child reports (*d* = 0.63, *p* < .001) and large effect size in parent reports (*d* = 0.81, *p* < .001). Clinically significant improvement (>30%) in gastrointestinal symptoms was achieved by 40% of completers. Pain intensity showed a modest decrease, with small effect sizes in both child (*d* = 0.24, *p* < .005) and parent reports (*d* = 0.28, *p* < .005).

**Conclusion:**

The Swedish version of audio-based gut-directed hypnotherapy appears feasible and acceptable, with promising symptom improvements in children with functional gastrointestinal disorders. A randomized controlled trial should be conducted to confirm efficacy and identify predictors of treatment response.

## Plain language summary

Hypnotherapy using Audio Exercises for Pediatric Functional Abdominal Pain Disorders: A Swedish Pilot Study

Gut-directed hypnotherapy is a treatment that uses relaxation and imagination exercises to help children and adolescents manage symptoms of functional gastrointestinal disorders, such as irritable bowel syndrome (IBS), functional abdominal pain, and functional dyspepsia. While this approach has been effective in other countries, it has not been previously available in pediatric healthcare in Sweden.

This study aimed to test whether a Swedish version of this treatment, delivered through audio recordings, would be feasible and beneficial. The study involved 32 children and adolescents aged 8 to 17 years, who participated in a 12-week online hypnotherapy program, adapted from a validated Dutch protocol. Participants and their parents completed questionnaires before, during, and after the program to assess gastrointestinal symptoms, pain and psychological symptoms.

The results were promising. Most participants completed the program, and both children and their parents reported significant improvements in gastrointestinal symptoms. Pain intensity also decreased, though to a lesser extent. Additionally, the treatment was well-received, with adequate levels of credibility and satisfaction.

This study suggests that audio-based gut-directed hypnotherapy is a feasible and acceptable treatment option for children with functional gastrointestinal disorders in Sweden. It offers a cost-effective and accessible alternative to traditional therapies. A larger randomized controlled trial is now needed to confirm these findings and identify which patients benefit most from this treatment.

## Introduction

Disorders of gut-brain interaction (DGBI), including irritable bowel syndrome (IBS), functional abdominal pain-not otherwise specified (FAP-NOS), and functional dyspepsia (FD), are common among children and adolescents worldwide.^1^ In Sweden, the prevalence of these conditions ranges from 10% to 25%, depending on age and sex.^2^ Despite the benign nature of these conditions, these patients often suffer significantly from their symptoms and have a diminished quality of life,^3^ increased school absenteeism,^4^ and a high demand for healthcare resources.^5,6^

The diagnosis is confirmed when the Rome IV criteria are met if, after appropriate medical evaluation, the symptoms cannot be attributed to another medical condition.^7^ Confirming the diagnosis and ensuring that patients and their caregivers understand the mechanisms underlying these disorders is a crucial first step in treatment.^8^ For many patients, this alone may be sufficient to alleviate symptoms, as previously shown for adult IBS patients.^9^ Although pharmacological treatments—such as anti-spasmodics, anti-depressants, anti-emetics, and proton pump inhibitors—are frequently used, the evidence supporting their efficacy in managing DGBI in the pediatric population is limited or lacking.^10^

The treatment approaches that have been proven effective primarily involve behavioral interventions, with cognitive behavioral therapy (CBT) and hypnosis showing the most promising results.^11,12^ These modalities are also increasingly being delivered as digital therapies to meet the growing demand.^13^ For children and adolescents with DGBI in Sweden, exposure-based CBT is the most widely used psychological treatment. It focuses on exercises to help reduce the avoidance behaviors that often both result from and contribute to the persistence of gastrointestinal symptoms.^14–17^ Currently, CBT is also available as an internet-based therapy (iCBT) for children and adolescents in Sweden, improving cost-effectiveness and accessibility across the country.^18–20^ However, the iCBT patients still require continuous support from an individual therapist, leading to long waiting times for this treatment. Moreover, exposure-based treatment does not work for all patients, indicating a need for alternative approaches that target different behaviors or underlying mechanisms, and are preferably even more accessible and cost-effective.

Medical gut-directed hypnotherapy (HT), developed in the 1980s (the Manchester method), has since been widely utilized to treat abdominal pain.^21,22^ The goal is to help patients develop tools that they can use independently to achieve a more relaxed state and gain better control when symptoms arise.^23,24^ The therapy involves an initial induction to guide the patient into a deeply relaxed hypnotic state. During this state, the patient is given suggestions and visualizations designed to alter their physical symptoms, feelings, thoughts, and behaviors related to their gastrointestinal symptoms.

Randomized controlled trials have demonstrated that HT is highly effective for treating pediatric DGBI in both the short and long term, when compared to standard medical care.^24,25^ HT has also been successfully used in Sweden with adult IBS patients using both individual and group HT sessions,^26,27^ with very promising long-term effects.^28^ Despite the strong evidence in adult care in Sweden and internationally for the pediatric population, HT is not currently available as a treatment option for children with DGBI within the Swedish public healthcare.

Multiple studies have demonstrated that hypnotherapy can be effectively administered as a home-based self-guided intervention through the use of digital platforms.^29–32^ The 12-week self-guided HT program for children and adolescents used in this study has been evaluated in a large randomized controlled trial in the Netherlands and shown to be non-inferior to six sessions of face-to-face delivered HT.^33^ The positive outcomes of audio-based HT have been further supported by long-term follow-up studies, and ongoing implementation research in the Netherlands is currently exploring its integration into routine clinical practice.^34–36^

This study represents the first pilot phase of a larger project aimed at establishing and evaluating home-based gut-directed HT as a treatment option for children and adolescents with DGBI in Sweden. The original Dutch treatment protocol has been translated and culturally adapted for Swedish children. In this pilot study, we aimed to evaluate feasibility, including practical aspects such as patient recruitment and effect sizes in the sample, in preparation for a larger randomized controlled trial.

## Patients and methods

### Design

A feasibility study that measures the within-group effect of HT self-exercises delivered by audio files in a pre-post design. This study was registered in clinicaltrials.gov (NCT06493097) and conducted in accordance with the Helsinki Declaration of 1975 as revised in 2013. This study was conducted and reported by the CONSORT 2010 extension for pilot and feasibility trials guidelines^37^ (Supplementary Table 1).

### Participants

Children and adolescents with DGBI aged 8–17 years at the start of the treatment were recruited from Sachs Children’s Hospital and four outpatient pediatric clinics in Stockholm between October 2023 and June 2024. We aimed to include a minimum of 30 participants in the study, based on previous recommendations of sample size in pilot studies.^38^ All patients underwent a physical examination and a basic laboratory work-up by a physician in the study team before inclusion. Written informed consent was obtained from the parents of children under 15 years and from adolescents over 15 years of age. Verbal informed consent was obtained from all participants. All patient details were de-identified in the text, figures, and tables.

Inclusion criteria were (1) IBS, FAP-NOS, or FD diagnosis according to the Rome IV criteria, (2) in case of psychopharmacological medication, stable dose at least 2 months before study start, and (3) adequate and stable pharmacological regimen for constipation in case of constipation-predominant IBS for a minimum of one month before study initiation.

Reasons for exclusion were (1) other nonfunctional medical conditions that better explained the symptoms, (2) other circumstances such as ongoing psychological treatment or >40% school absenteeism, that are considered to require more intensive interventions than self-administered HT, and (3) lack of required Swedish language skills.

### Treatment protocol

HT treatment was based on the protocol originally described in the study by Rutten et al.^33^ The protocol was initially translated into Swedish by professional translators. Subsequently, two native Swedish-speaking therapists, specialized in HT, independently reviewed the translation and harmonized their versions into a final, culturally adapted version. These adaptations made to the Dutch protocol were minimal, limited to wording adjustments, and did not alter the substantive content. The texts were recorded in a studio with audiobook quality. A web platform (BASS, Karolinska Institutet) was used to distribute the HT treatment as well as to collect the data with internationally validated symptom scales.

The treatment lasted for 12 weeks and consisted of five HT audio files, each approximately 15 min long, tailored to two age groups: 8–12 years and 13–17 years. Participants were instructed to listen to the audio files at least five times per week, following the guidelines provided on the website. The audio files could be downloaded to a mobile device. The online platform included a logbook where participants were asked to report their weekly exercise frequency. The platform also included informational materials, in both text and film format, designed to introduce the treatment mechanisms at an age-appropriate level.

### Feasibility criteria

One key feasibility criterion for planning the future randomized controlled trial (RCT) was the recruitment rate and timeline. A feasible inclusion rate at a single center was defined as one patient per week, suggesting that nationwide recruitment could ensure a steady and sufficient participant flow. In addition, feasibility was evaluated considering drop-out rate, treatment adherence, participant satisfaction and acceptability, completeness of outcome data, time for intervention delivery, appropriateness of the assessment procedures, and effect size for the primary outcome in the sample.

### Measurements

Participants and their parents completed a series of questionnaires at multiple time points: before the treatment began, every 3 weeks during treatment, and after its completion. Some questionnaires were administered at all five time points, while others were completed only at pre- and post-treatment assessments, or just once during the treatment period. A full list of the questionnaires used, and their administration time points, is provided in Supplemental Table 2.

*Treatment credibility* was evaluated by children and parents at week 3, using a 4-item child-adapted version of the *Credibility Scale*, which assesses both the perceived credibility of the treatment and the expectancy of improvement.^39^ Each question has a continuous scale of 0–10, and the total score for each patient was calculated as the average score of all 4 items.

*Treatment satisfaction* was measured from children and parents after the treatment using an 8-item *Client Satisfaction Scale* (CSQ-8) with an answer range 1–4 (1 = bad, 4 = very good), giving a total point range 8–32, where a higher score indicates higher satisfaction.^40^

*Adequate relief* was assessed by children and parents after the treatment with the *Subject’s Global Assessment of Relief* (SGA), where symptoms after treatment compared to pretreatment are evaluated with one question rated 0 (=worsened a lot) to 6 (=improved a lot).^41^

*The Pediatric Quality of Life Inventory Gastrointestinal Symptom Scale (PedsQL Gastro)* is a 9-item short scale evaluating gastrointestinal symptoms, including pain and discomfort, abnormalities in stool consistency, nausea and vomiting, loss of appetite, discomfort, flatulence, and bloating during the last month. The scale 0–100 is inverted so that a higher score indicates fewer symptoms.^42^ PedsQL Gastro questionnaire was measured at each time point from both children and parents. A clinically significant effect in this primary outcome was defined as a 30% or greater improvement, as previously recommended in studies of chronic pain.^16,17,43,44^

*Pain intensity* was measured using the *Faces Pain Scale Revised*, which features drawn facial expressions representing pain levels from 0 (no pain) to 10 (worst pain).^45^ Patients and caregivers used this scale to rate the intensity of pain experienced at its worst during the past week before reporting. *Pain frequency* was assessed by reporting the number (0–7) of days with the worst pain during the previous week. Pain intensity and frequency were measured at each time point by children and parents.

*The Pediatric Quality of Life Inventory* *(Peds-QL QOL)* was used to assess quality of life at pre- and post-treatment by both children and parents. Gastrointestinal avoidance and anxiety were measured using child-adapted short scales from the *Irritable Bowel Syndrome-Behavioral Responses Questionnaire* (BRQ) and the *Visceral Sensitivity Index* (VSI), respectively.^46,47^ Stress *(Pressure Activation Stress, PAS)*, anxiety (*Spence Children Anxiety Scale, SCAS*), and depression (*Children’s Depression Inventory, CDI*) were also measured, as these are known common comorbidities of DGBI.^48,49^ Anxiety and depression were evaluated at pre- and post-measurements, whereas the stress, avoidance behavior, and GI-anxiety were included in questionnaires at each time point. All these questionnaires were solely answered by the children.

*School absence* was reported pre- and post-treatment by parents in days of school absence during the last 3 weeks before measurement.

### Statistical analyses

Measurements that were obtained before, during, and after treatment were analyzed using linear mixed models (LMM). The 12-week effect of the hypnotherapy treatment was calculated by multiplying the weekly effect of the treatment by 12. To obtain Cohen’s *d* effect size, the 12-week treatment effect was divided (standardized) by the standard deviation (SD) at baseline. A paired t-test was conducted to evaluate the statistical significance (*p*-value) of the mean difference between pre- and post-intervention measurements

The outcome variables that only had pre- and post-measurements were analyzed with Cohen’s adjusted *d* for paired data together with Hedges’ small sample bias correction.^50^

To avoid overestimating the treatment effect, a sensitivity analysis comparing the results of removing the missing values to imputing the missing values using Last Observation Carried Forward was performed.

All statistical analyses were performed using the R Studio software, version 4.5.0.

As there are no established thresholds for credibility, satisfaction, or adequate relief scores, we interpret findings based on the relative position of scores within the scale range and the overall pattern of responses within the sample. The mean score for credibility and the total score (sum) for satisfaction for all items were calculated.

## Results

### Patient flow and compliance

**Figure 1.**
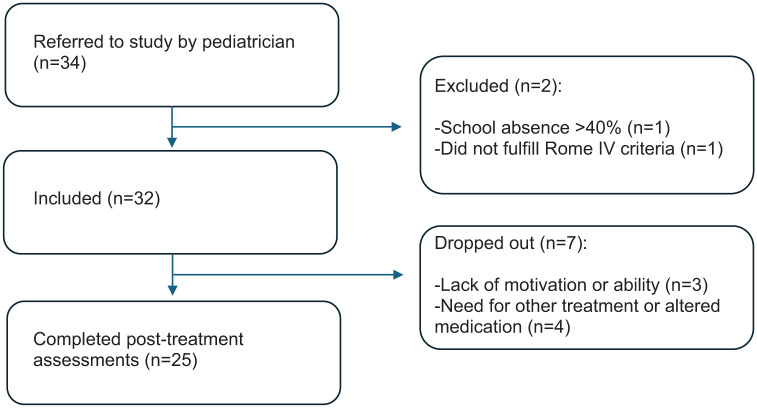
Participant flow in the study.

Of the 34 patients referred to the study, two patients were excluded for not meeting the inclusion/exclusion criteria, and 32 were enrolled in the study (Figure 1). Characteristics of the included patients are listed in Table 1. The post-treatment questionnaire was answered by 25/32 (78%) patients. The number of patients who dropped out was 7/32 (22%), none of whom replied to the post-treatment questionnaires. Reasons for discontinuation included participant-reported factors, such as perceiving the treatment as excessively time-consuming or experiencing difficulty concentrating on the exercises, as well as medical reasons, including changes in medication that could impact study outcomes or the initiation of other psychological treatments during the study period.

**Table 1.** Patient characteristics at baseline.

Age (years), mean [range]	12.6 [8–17]
Age, 8–12 y *N* (%)	16 (50)
Age, 13–17 y *N* (%)	16 (50)
Sex, male *N* (%)	10 (31)
Sex, female *N* (%)	22 (69)
Diagnosis, IBS *N* (%)	22 (68)
Diagnosis, FAP *N* (%)	5 (16)
Diagnosis, FD *N* (%)	5 (16)
Symptom duration >6 months *N* (%)	32 (100)
Symptom duration >1 year *N* (%)	27 (84)
Symptom duration >2years *N* (%)	24 (75)

FAP, functional abdominal pain; FD, functional dyspepsia; IBS, irritable bowel syndrome.

### Adherence to treatment protocol

Adherence was measured by manual logbooks via the website, which patients were asked at inclusion to fill out every week during the treatment. According to these logbooks, 9 (28%) of the 32 included patients completed the full treatment, reporting a minimum of 5 exercises per week during the whole 12-week period. Nearly half of the patients (15 (47%)) reported a total of 9–11 weeks of exercising a minimum of 5 times per week in the logbooks.

### Treatment credibility and its association with treatment outcome

**Figure 2.**
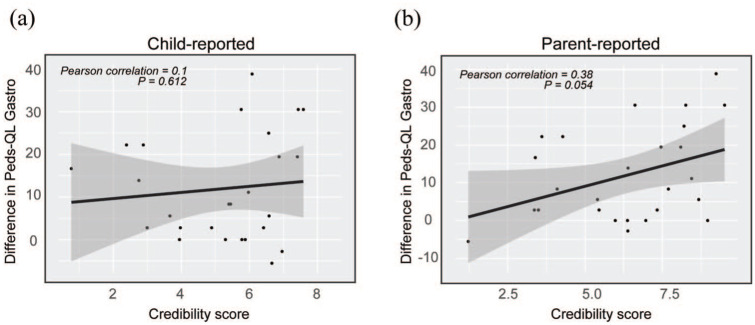
Treatment credibility versus difference in pre-post treatment gastrointestinal symptoms. (a) Individual child-reported credibility score in correlation to the difference (week 12-week 0) in child-reported PedsQL-gastro score. (b) Individual parent-reported credibility score in correlation to the difference (week 12–week 0) in child-reported PedsQL-gastro score.

The credibility questionnaire, mirroring the expectations of treatment success at week 3, was answered by 30/32 (94%) of patients, including 5 of the patients who dropped out later. Both children and parents rated the credibility as intermediate level in the mean score for all participants. Child-rated credibility was slightly lower (mean 5.2; range 0.8–8.3) than parent-rated credibility (mean 6.2; range 1.2–9.5). A total of 60% (18/30) of children rated the intervention credible or highly credible (= average score of all questions >5), compared to 67% of parents (20/30). Child-rated credibility did not show a significant linear correlation with improvement in the primary outcome, the child-rated PedsQL Gastro score (Pearson *r* = 0.1, *p* = 0.612; Figure 2(a)). In contrast, parents’ expectations demonstrated a moderate linear correlation with the child-rated treatment outcome, although not reaching statistical significance (Pearson *r* = 0.38, *p* = 0.054; Figure 2(b)).

### Treatment satisfaction

**Figure 3.**
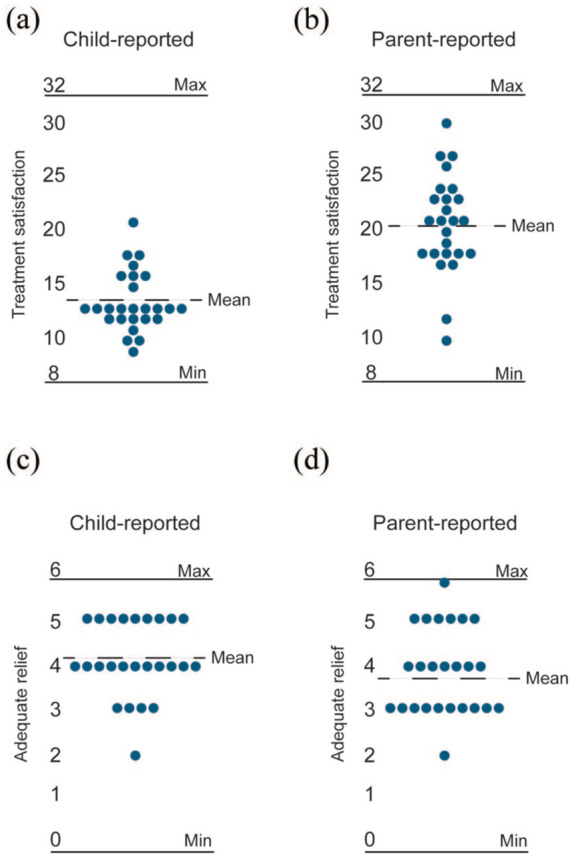
Treatment satisfaction and adequate relief. (a) Child-reported satisfaction with treatment at week 12 (scale range 8-32). Mean 13.7. (b) Parent-reported satisfaction with treatment at week 12 (scale range 8–32). Mean 20.7. (c) Child-reported adequate relief week 12 (scale range 0–6). Mean 4.12. (d) Parent-reported adequate relief week 12 (scale range 0–6). Mean 3.84.

The treatment satisfaction questionnaire (CSQ-8) was completed by children and parents at week 12 (post-treatment). Children scored the treatment satisfaction lower (mean 13.7; range 9–21, Figure 3(a)) than parents (mean 20.7; range 10–30; Figure 3(b)). While 60% (15/25) of parents rated their satisfaction on the positive side of the scale (= total score over 20), only 4% (1/25) of the children did so. In the assessment of adequate relief (SGA), however, children reported a slightly higher (mean 4.12, range 2–5; Figure 3(c)) score than parents (mean 3.84; range 2–6; Figure 3(d)). A total of 80% (20/25) of the children rated the adequate relief as positive (=score >3), whereas 52% (13/25) of the parents did so.

### Primary outcome: Gastrointestinal symptoms

**Figure 4.**
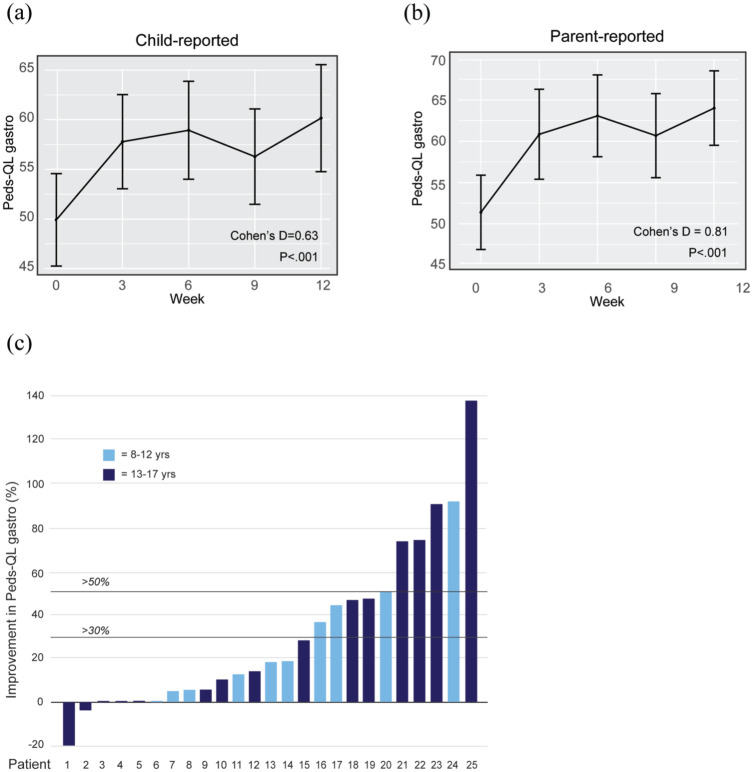
Gastrointestinal symptoms. (a) Mean child-reported pedsQL gastro per week of treatment. (b) Mean parent-reported pedsQL gastro per week of treatment. (c) Difference in child-reported pedsQL gastro by individual patients from week 0 (pre-) to week 12 (post-treatment). The PedsQL-gastro scale is inverted, so higher points indicate fewer symptoms.

A significant improvement in gastrointestinal symptoms (PedsQL Gastro) was observed from over the measurement period, as reported by both the patients [moderate effect size (*d* =0.63, *p* < 0.001, 95% CI 0.36–0.9)] and their parents [large effect size (*d* = 0.81, *p*<.001, 95% CI 0.45–1.17)] regarding their child’s symptoms (Figure 4(a) and (b), Table 2). A clinically significant improvement, defined as a >30% increase in the PedsQL Gastrointestinal Symptoms Scale score, was achieved by 40% of the patients who completed the post-treatment assessment; of these, 6 out of 25 patients (24%) achieved an improvement of 50% or more (Figure 4 (c)).

**Table 2.** Estimated means and effect sizes for children’s and parents’ estimates from pre-treatment baseline to the 12-week post-treatment evaluation.

Scale	Pre-mean (SD)	Post-mean (SD)	Cohen’s *d* (95% CI)	*p*-Value
Child reported				
PedsQL gastro	49.91 (13.43)	60.15 (14.01)	0.63 (0.36, 0.9)	0.000***
Faces	5.69 (2.28)	4.54 (2.3)	0.24 (−0.07, 0.55)	0.002**
Pain frequency	2.75 (1.48)	2.27 (1.82)	0.04 (−0.53, 0.55)	0.54
PedsQL QOL	70.48 (11.68)	73.54 (15.59)	0.19 (−0.14, 0.53)	0.12
BRQ-C	35.59 (13.38)	35.65 (13.64)	0.15 (−0.08, 0.38)	0.11
VSI-C	17.38 (9.14)	16.56 (8.22)	0.17 (−0.11, 0.45)	0.17
PAS-C	15.78 (10.33)	19.4 (11.15)	0.13 (−0.04, 0.34)	0.16
CDI-S	3.31 (3.53)	3.81 (3.2)	0.01 (−0.21, 0.36)	0.95
SCAS-C	14.75 (8.76)	15.65 (9.46)	0.01 (−0.17, 0.18)	0.94
Parent reported				
PedsQL gastro	51.39 (12.97)	64 (11.49)	0.81 (0.45, 1.17)	0.000***
Faces	5.69 (2.33)	4.48 (2.73)	0.28 (−0.08, 0.65)	0.016*
Pain frequency	3.16 (1.63)	2.16 (1.86)	0.31 (−0.14, 0.79)	0.09
PedsQL QOL	72.11 (12.39)	73.54 (15.59)	0.18 (−0.11, 0.49)	0.25
School absence	0.81 (1)	0.6 (0.91)	0.22 (−0.05, 0.45)	0.13

BRQ-C, Behavioral Responses Questionnaire short scale; CDI-S, Children’s Depression Inventory; Faces, Faces Pain Rating Scale; PAS-C, Pressure-activation-stress scale short version; PedsQL-gastro, Pediatric Quality of Life Inventory Gastrointestinal Symptom Scale; PedsQL QOL, Pediatric Quality of Life Inventory; SCAS, Spence Children Anxiety Scale Short version; VSI-C, Visceral Sensitivity Index short scale.

*p*-value *<0.05, **<0.01, ***<0.001.

### Pain intensity and frequency

**Figure 5.**
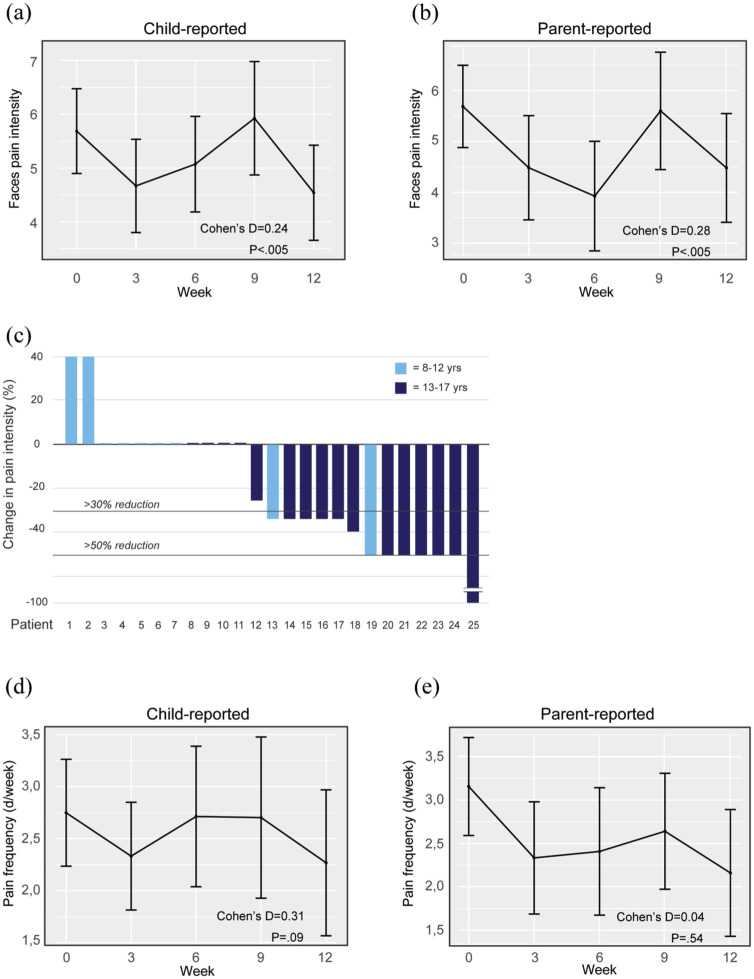
Pain intensity and pain frequency. (a) Child-reported pain intensity (Faces scale; pain at its worst) per week of treatment. (b) Parent-reported pain intensity (Faces scale) per week of treatment. (c) Difference in child-reported pain intensity (Faces scale) from week 0 (pre-) to week 12 (post-treatment). (d) Child-reported pain frequency (number of days in a week with pain at its worst) per week of treatment. (e) Parent-reported pain frequency (number of days in a week with pain at its worst) per week of treatment.

Pain intensity reduction over the entire treatment period showed small to moderate effect sizes in both child-reported (*d* = 0.24, 95% CI −0.07, 0.55) and parent-reported data (*d* = 0.28, 95% CI −0.08, 0.65) (Figure 5(a) and (b), Table 2). Although the confidence intervals span zero—likely reflecting variability across time points and a nonlinear progression of symptom change—the overall reduction in pain intensity from pre- to post-treatment was statistically significant at the group level for both child- (*p* = 0.002) and parent-rated measures (*p* = 0.015), as indicated by a paired *t*-test. A clinically significant reduction in pain intensity (defined as >30% reduction) was observed in 13 out of 25 patients (52%) based on child-reported measures, and a 50% or more reduction in pain intensity was achieved by 7/25 (28%) patients (Figure 5(c)).

Although numerically decreased, we did not observe any statistically significant change in mean pain frequency between pre- and post-treatment measurements in children’s or parents’ reports (Figure 5(d) and (e), Table 2).

### Other outcome measurements

For other outcome measurements (e.g., quality of life, visceral sensitivity, avoidance behavior, stress, depression, anxiety, and school absence), we did not observe any statistically significant changes in pre- versus post-treatment measurements in this cohort (Table 2).

## Discussion

### Feasibility

The measured feasibility in terms of patient recruitment rate, data collection, parents’ satisfaction with treatment, and the effect sizes of the primary outcomes met the predefined expectations and criteria for proceeding to a larger trial. However, not all feasibility criteria were met; specifically, the satisfaction score of the children was in the lower range of the scale, and the study faced challenges with suboptimal adherence to the treatment protocol, as recorded in the manual logbooks.

### Attrition and adherence

The drop-out rate in this study at 22%, which was in line with rates observed in other self-administered psychological interventions.^51^ Interestingly, all drop-outs occurred in the 8–12-year-old age group. This may indicate that, despite age-appropriate adjustments to the protocol, self-guided treatment of this intensity poses greater challenges for younger children. In this age range, responsibility for adherence to the protocol largely falls on caregivers.

Only one-third of the participants reported having completed the full 12-week program. On the other hand, 75% of participants completed at least 9 out of the 12 weeks (75%) of the intervention, which represents a strong level of engagement for a self-guided program and may still confer meaningful therapeutic benefits. Although suboptimal compared to the Dutch studies with the same protocol, this level of adherence rate is considered acceptable for many other self-administered psychological interventions.^32,52,53^ Maintaining motivation from the outset is crucial for both children and their caregivers to ensure treatment success. Motivation could potentially be enhanced through improvements to the usability and visual appeal of the treatment platform, as well as by implementing default support strategies such as proactively contacting all participating families during treatment, an approach successfully used in the Dutch study protocol.^34^

In addition to the treatment intensity, the study protocol included frequent assessment points and a comprehensive set of questionnaires, which may have contributed to participant fatigue—both in children and their parents—and negatively impacted adherence to the protocol. For this reason, future studies should carefully consider the selection of outcome measures and timepoints to minimize participant burden and reduce the risk of drop-out.

### Treatment credibility and satisfaction

The average treatment credibility after 3 weeks was in the neutral range of the scale, although parents rated it higher than children and adolescents. Treatment credibility has been shown to correlate with outcome, adherence, and drop-out rate in online-delivered psychotherapy programs for adults.^54^ In this study, the parents’ credibility ratings also demonstrated a correlation with treatment outcome, possibly indicating that their expectations and biases may influence their child’s motivation—and that knowledge of their own child’s characteristics may allow them to anticipate the treatment’s likely effectiveness and suitability. The moderate credibility scores might indicate the need for more thorough explanations of the treatment mechanisms and the potential for improvement to the children and adolescents, particularly given that the term “hypnotherapy” may evoke certain misconceptions.

Children rated the treatment satisfaction (CSQ-8) in the lower range of the scale, and notably lower than parents. This questionnaire includes items such as “How did you like this treatment?” and “Would you recommend this treatment to a friend?”. In contrast, the adequate relief- answering the question “How well did this treatment help you with your problems?”—was rated higher by the children than the parents, and both rated it above the mid-range, indicating a positive response. This discrepancy in children’s ratings of the two scales may suggest that, although the children perceived the treatment as beneficial, they also found it demanding and time-consuming.

### Main results and interpretation

For the primary outcome, PedsQL Gastro Short Scale measuring gastrointestinal symptoms, we observed a moderate within-group effect size on child-reported and a large effect size on parent-reported difference in means. Treatment response varied considerably within the group, where 40% of patients who underwent the treatment achieved clinically significant improvement (defined as >30% improvement in PedsQL gastro). Identifying individuals who are more likely to benefit from this intervention would be valuable in guiding personalized treatment choices and optimizing care. In comparison to another remotely delivered treatment, iCBT, evaluated in an RCT of 8–12-year-old children by Lalouni et al.,^19^ the within-group effect size for the PedsQL Gastro was significantly larger (*d* = 1.11, 95% CI: 0.82–1.42) than that observed in the present study using the HT audio file protocol. However, direct comparison between studies should be made with caution, given differences in study populations.

We also observed a significant reduction in pain intensity, as reported by both children and parents using the Faces Pain Scale—one of the secondary outcomes in this study. In comparison, the Dutch RCT by Rutten et al.^33^ found that over 60% of participants who received HT treatment via audio files using a similar protocol experienced a 50% reduction in pain intensity, which was the primary outcome in that study.^34^ In this current study, the percentage of patients experiencing a 50% reduction in pain intensity was only 22%. Several factors may explain the differences in outcomes between the two studies. Participants in the present study were primarily recruited from a tertiary care center, had a longer duration of symptoms, and many had previously tried other treatments without success. This may suggest that the intervention is more effective when initiated earlier in the course of the condition, and in fact, introducing HT as an early intervention is supported by previous studies.^55^ Differences in how the treatment was administered may also have influenced outcomes—for example, variations in delivery format, guidance, and follow-up during the treatment could affect engagement and efficacy. In addition, direct comparison is complicated by differences in outcome measures and pain assessment methods, specifically, the use of daily pain diaries in previous studies versus weekly ratings of worst pain in the current study. Overall, to accurately evaluate the relative effectiveness of different interventions, it is crucial to harmonize outcome measures and study populations—ideally through direct, head-to-head comparisons in randomized controlled trials.^15^

In this relatively small cohort, no significant changes were observed in the parameters measuring quality of life, depression, anxiety, gastrointestinal symptom-specific anxiety, stress, or avoidance behavior (Table 2), in contrast to the larger Dutch cohort, where improvements were also seen in depression, anxiety, and somatization scores, quality of life, and pain beliefs.^34^ Specifically, stress could have been expected to decrease, since the mechanism of HT is previously connected to stress reduction.^56^ The lack of effect in avoidance behavior and gastrointestinal anxiety (BRQ and VSI scales) was more expected as the treatment is not aimed at reducing these symptoms, in contrast to CBT.^15^

### Strengths and limitations

The strengths of this study are testing a novel treatment modality for DGBI, previously not available in Sweden, as well as a comprehensive and frequent data collection with several outcome measurements throughout the treatment period. A within-group design was chosen to serve the purpose of testing the feasibility of the treatment and the study protocol, usability of the digital platform and data collection process, and to assess the within-group effect sizes to guide the design and calculate power for a large RCT. The within-group study design is a limitation, as true efficacy cannot be assessed while regression toward the mean cannot be controlled for. Another limitation was the lack of reliable compliance reporting, which could have been addressed by restricting access to the audio files solely through the website. However, we chose to allow patients to download the files to make the treatment as accessible and convenient as possible and report manually, which many patients failed to do as instructed.

## Conclusion and future aspects

This study demonstrates that the Swedish version of HT self-exercises using audio files is a feasible treatment option for children and adolescents with IBS, FD, or FAP-NOS, and may be effective in reducing gastrointestinal symptoms and pain.

Based on the findings of this study, an improved, automated method for monitoring adherence through the web-based platform is needed. In addition, incorporating a brief human interaction midway through the intervention may help enhance motivation and support continued engagement with the treatment.

An important factor in predicting treatment outcomes and selecting suitable candidates for HT is the individual’s psychological and somatic symptom profile. For example, previous research in adults with IBS suggests that those with a higher gastrointestinal symptom burden and lower levels of anxiety and depression tend to respond more favorably to HT.^57^ Exploring whether similar patterns apply to pediatric populations could be valuable in future studies. Equally important is evaluating which patients are likely to benefit from and adhere to a self-administered, home-based treatment format. While HT using audio files is likely cost-effective and easy to disseminate, it may not be appropriate for all patients, like those with neurodiversity or limited support from caregivers in managing the treatment. Subgroup analyses exploring these factors will be of significant value in a larger study cohort. Ideally, professionals like nurses could be trained to give group or individual face-to-face hypnotherapy, or a hybrid model that combines in-person and remote approaches could be considered for implementation in Swedish pediatric healthcare, as has been done for adult patients.^27,58^

An RCT with a larger sample size is needed to confirm these preliminary findings, directly compare the efficacy of self-administered HT with other established treatments such as exposure-based CBT, and identify patient subgroups most likely to benefit from this approach. Furthermore, a qualitative study involving interviews with participants and their caregivers could provide valuable insights into practical improvements for the treatment platform, including usability, user experience, credibility, and factors influencing adherence.

## Supplemental Material

sj-doc-1-tag-10.1177_17562848251381141 – Supplemental material for Hypnotherapy self-exercises with audio files for children and adolescents with disorders of gut-brain interaction—a study of feasibility and preliminary effects in Sweden Supplemental material, sj-doc-1-tag-10.1177_17562848251381141 for Hypnotherapy self-exercises with audio files for children and adolescents with disorders of gut-brain interaction—a study of feasibility and preliminary effects in Sweden by Tea Soini, Frida Andersson, Maria Lalouni, Marianne Bonnert, Siri Voghera, Brjánn Ljótsson, Marc A. Benninga, Arine M. Vlieger, Helena J. Rolandsdotter, Agneta Uusijärvi and Ola Olén in Therapeutic Advances in Gastroenterology

sj-docx-2-tag-10.1177_17562848251381141 – Supplemental material for Hypnotherapy self-exercises with audio files for children and adolescents with disorders of gut-brain interaction—a study of feasibility and preliminary effects in Sweden Supplemental material, sj-docx-2-tag-10.1177_17562848251381141 for Hypnotherapy self-exercises with audio files for children and adolescents with disorders of gut-brain interaction—a study of feasibility and preliminary effects in Sweden by Tea Soini, Frida Andersson, Maria Lalouni, Marianne Bonnert, Siri Voghera, Brjánn Ljótsson, Marc A. Benninga, Arine M. Vlieger, Helena J. Rolandsdotter, Agneta Uusijärvi and Ola Olén in Therapeutic Advances in Gastroenterology

## Declarations

**Ethics approval and consent to participate:** This study was approved by the ethical review committee in Stockholm (reference numbers 2023-00296-01, dated 18th April 2023, and 2023-04312-02, dated 5th September 2023). Written informed consent was obtained from the parents of children under 15 years and from adolescents over 15 years of age. Verbal informed consent was obtained from all participants.

**Consent for publication:** Not applicable for this study.

## References

[1] Koen Vermeijden N de Silva L Manathunga S , et al. Epidemiology of pediatric functional abdominal pain disorders: a meta-analysis. Pediatrics 2025; 155(2): e2024067677. 10.1542/peds.2024-067677 39761807

[2] Sjölund J Uusijärvi A Tornkvist NT , et al. Prevalence and progression of recurrent abdominal pain, from early childhood to adolescence. Clin Gastroenterol Hepatol 2021; 19(5): 930–938.e8. 10.1016/j.cgh.2020.04.047 32344151

[3] Sjölund J Kull I Bergström A , et al. Quality of life and bidirectional gut-brain interactions in irritable bowel syndrome from adolescence to adulthood. Clin Gastroenterol Hepatol 2024; 22(4): 858–866.e6. 10.1016/j.cgh.2023.09.022 37802270

[4] Tersteeg SM Borowitz SM. School absenteeism as a predictor of functional gastrointestinal disorders in children. Front Pediatr 2024; 12: 1503783. 39726527 10.3389/fped.2024.1503783 PMC11669597

[5] Dhroove G Chogle A Saps M. A million-dollar work-up for abdominal pain: is it worth it? J Pediatr Gastroenterol Nutr 2010; 51(5): 579–583. 20706149 10.1097/MPG.0b013e3181de0639

[6] Uusijärvi A Olén O Malmborg P , et al. Combining Rome III criteria with alarm symptoms provides high specificity but low sensitivity for functional gastrointestinal disorders in children. Acta Paediatr 2018; 107(9): 1635–1641. 29486063 10.1111/apa.14297

[7] Hyams JS Di Lorenzo C Saps M , et al. Functional disorders: children and adolescents. Gastroenterology 2016: S0016-5085(16)00181-5. 10.1053/j.gastro.2016.02.015 27144632

[8] Schechter NL Coakley R Nurko S. The golden half hour in chronic pediatric pain-feedback as the first intervention. JAMA Pediatr 2021; 175(1): 7–8. 32628256 10.1001/jamapediatrics.2020.1798

[9] Ringström G Störsrud S Posserud I , et al. Structured patient education is superior to written information in the management of patients with irritable bowel syndrome: a randomized controlled study. Eur J Gastroenterol Hepatol 2010; 22(4): 420–428. 19923998 10.1097/MEG.0b013e3283333b61

[10] Rexwinkel R De Bruijn CMA Gordon M , et al. Pharmacologic treatment in functional abdominal pain disorders in children: a systematic review. Pediatrics 2021; 147: e2020042101. 10.1542/peds.2020-042101 34045320

[11] Axelsson E Kern D Hedman-Lagerlöf E , et al. Psychological treatments for irritable bowel syndrome: a comprehensive systematic review and meta-analysis. Cogn Behav Ther 2023; 52(6): 565–584. 37341454 10.1080/16506073.2023.2225745

[12] Sinopoulou V Groen J Gordon M , et al. Efficacy of interventions for the treatment of irritable bowel syndrome, functional abdominal pain—not otherwise specified, and abdominal migraine in children: a systematic review and network meta-analysis. Lancet Child Adolesc Health 2025; 9(5): 315–324. 40246358 10.1016/S2352-4642(25)00058-6

[13] Brenner DM Ladewski AM Kinsinger SW. Development and current state of digital therapeutics for irritable bowel syndrome. Clin Gastroenterol Hepatol 2024; 22(2): 222–234. 37743035 10.1016/j.cgh.2023.09.013

[14] Bonnert M Olén O Bjureberg J , et al. The role of avoidance behavior in the treatment of adolescents with irritable bowel syndrome: a mediation analysis. Behav Res Ther 2018; 105: 27–35. 29614378 10.1016/j.brat.2018.03.006

[15] Lalouni M Hesser H Bonnert M , et al. Breaking the vicious circle of fear and avoidance in children with abdominal pain: a mediation analysis. J Psychosom Res. Epub ahead of print January 2021. DOI: 10.1016/j.jpsychores.2020.110287. 33227558

[16] Lalouni M Ljótsson B Bonnert M , et al. Internet-delivered cognitive behavioral therapy for children with pain-related functional gastrointestinal disorders: feasibility study. JMIR Ment Health 2017; 4(3): e32. 10.2196/mental.7985 PMC5571236 28798012

[17] Bonnert M Olén O Lalouni M , et al. Internet-delivered exposure-based cognitive-behavioral therapy for adolescents with functional abdominal pain or functional dyspepsia: a feasibility study. Behav Ther 2019; 50(1): 177–188. 30661558 10.1016/j.beth.2018.05.002

[18] Bonnert M Olén O Lalouni M , et al. Internet-delivered cognitive behavior therapy for adolescents with irritable bowel syndrome: a randomized controlled trial. Am J Gastroenterol 2017; 112(1): 152–162. 27845338 10.1038/ajg.2016.503

[19] Lalouni M Ljótsson B Bonnert M , et al. Clinical and cost effectiveness of online cognitive behavioral therapy in children with functional abdominal pain disorders. Clin Gastroenterol Hepatol 2019; 17(11): 2236–2244.e11. 10.1016/j.cgh.2018.11.043 30502501

[20] Sampaio F Bonnert M Olén O , et al. Cost-effectiveness of internet-delivered cognitive-behavioural therapy for adolescents with irritable bowel syndrome. BMJ Open 2019; 9(1): e023881. 10.1136/bmjopen-2018-023881 PMC6347900 30679293

[21] Gonsalkorale WM. Gut-directed hypnotherapy: the Manchester approach for treatment of irritable bowel syndrome. Int J Clin Exp Hypn 2006; 54(1): 27–50. 16316882 10.1080/00207140500323030

[22] Whorwell PJ Prior A Faragher EB. Controlled trial of hypnotherapy in the treatment of severe refractory irritable-bowel syndrome. Lancet 1984; 2(8414): 1232–1234. 6150275 10.1016/s0140-6736(84)92793-4

[23] Miller V Whorwell PJ. Hypnotherapy for functional gastrointestinal disorders: a review. Int J Clin Exp Hypn 2009; 57(3): 279–292. 19459089 10.1080/00207140902881098

[24] Vlieger AM Menko-Frankenhuis C Wolfkamp SCS , et al. Hypnotherapy for children with functional abdominal pain or irritable bowel syndrome: a randomized controlled trial. Gastroenterology 2007; 133(5): 1430–1436. 17919634 10.1053/j.gastro.2007.08.072

[25] Vlieger AM Rutten JMTM Govers AMAP , et al. Long-term follow-up of gut-directed hypnotherapy vs. Standard care in children with functional abdominal pain or irritable bowel syndrome. Am J Gastroenterol 2012; 107(4): 627–631. 22310221 10.1038/ajg.2011.487

[26] Lindfors P Unge P Arvidsson P , et al. Effects of gut-directed hypnotherapy on IBS in different clinical settings-results from two randomized, controlled trials. Am J Gastroenterol 2012; 107(2): 276–285. 21971535 10.1038/ajg.2011.340

[27] Lövdahl J Törnblom H Ringström G , et al. Randomised clinical trial: individual versus group hypnotherapy for irritable bowel syndrome. Aliment Pharmacol Ther 2022; 55(12): 1501–1511. 35505463 10.1111/apt.16934 PMC9324196

[28] Lindfors P Unge P Nyhlin H , et al. Long-term effects of hypnotherapy in patients with refractory irritable bowel syndrome. Scand J Gastroenterol 2012; 47(4): 414–420. 22339617 10.3109/00365521.2012.658858

[29] van Tilburg MAL Chitkara DK Palsson OS , et al. Audio-recorded guided imagery treatment reduces functional abdominal pain in children: a pilot study. Pediatrics 2009; 124(5): e890–e897. 10.1542/peds.2009-0028 19822590

[30] Gulewitsch MD Müller J Hautzinger M , et al. Brief hypnotherapeutic-behavioral intervention for functional abdominal pain and irritable bowel syndrome in childhood: a randomized controlled trial. Eur J Pediatr 2013; 172(8): 1043–1051. 23568514 10.1007/s00431-013-1990-y

[31] Berry SK Berry R Recker D , et al. A randomized parallel-group study of digital gut-directed hypnotherapy vs muscle relaxation for irritable bowel syndrome. Clin Gastroenterol Hepatol 2023; 21(12): 3152–3159.e2. 10.1016/j.cgh.2023.06.015 37391055

[32] Peters SL Gibson PR Halmos EP. Smartphone app-delivered gut-directed hypnotherapy improves symptoms of self-reported irritable bowel syndrome: a retrospective evaluation. Neurogastroenterol Motil 2023; 35(4): e14533. 10.1111/nmo.14533 36661117

[33] Rutten JMTM Vlieger AM Frankenhuis C , et al. Gut-directed hypnotherapy in children with irritable bowel syndrome or functional abdominal pain (syndrome): a randomized controlled trial on self exercises at home using CD versus individual therapy by qualified therapists. BMC Pediatr 2014; 14: 140. 24894077 10.1186/1471-2431-14-140 PMC4060754

[34] Rutten JMTM Vlieger AM Frankenhuis C , et al. Home-based hypnotherapy self-exercises vs individual hypnotherapy with a therapist for treatment of pediatric irritable bowel syndrome, functional abdominal pain, or functional abdominal pain syndrome a randomized clinical trial. JAMA Pediatr 2017; 171(5): 470–477. 28346581 10.1001/jamapediatrics.2017.0091

[35] Rexwinkel R Bovendeert JFM Rutten JMTM , et al. Long-term follow-up of individual therapist delivered and standardized hypnotherapy recordings in pediatric irritable bowel syndrome or functional abdominal pain. J Pediatr Gastroenterol Nutr 2022; 75(1): 24–29. 35759537 10.1097/MPG.0000000000003478 PMC9236305

[36] Ganzevoort IN Fokkema T Mol-Alma HJ , et al. Home-based guided hypnotherapy for children with functional abdominal pain and irritable bowel syndrome in primary care: study protocol for a randomised controlled trial. BMJ Open 2023; 13(5): e069653. 10.1136/bmjopen-2022-069653 PMC10173965 37156587

[37] Eldridge SM Chan CL Campbell MJ , et al. CONSORT 2010 statement: extension to randomised pilot and feasibility trials. BMJ 2016; 355: i5239. 10.1136/bmj.i5239 PMC5076380 27777223

[38] Whitehead AL Julious SA Cooper CL , et al. Estimating the sample size for a pilot randomised trial to minimise the overall trial sample size for the external pilot and main trial for a continuous outcome variable. Stat Methods Med Res 2016; 25(3): 1057–1073. 26092476 10.1177/0962280215588241 PMC4876429

[39] Borkovec TD Nau SD. Credibility of analogue therapy rationales. J Behav Ther Exp Psychiatry 1972; 3(4): 257–260.

[40] Attkisson CC Zwick R. The client satisfaction questionnaire. Psychometric properties and correlations with service utilization and psychotherapy outcome. Eval Program Plann 1982; 5(3): 233–237. 10259963 10.1016/0149-7189(82)90074-x

[41] Müller-Lissner S Koch G Talley NJ , et al. Subject’s Global Assessment of Relief: an appropriate method to assess the impact of treatment on irritable bowel syndrome-related symptoms in clinical trials. J Clin Epidemiol 2003; 56(4): 310–316. 12767407 10.1016/s0895-4356(03)00027-1

[42] Varni JW Lane MM Burwinkle TM , et al. Health-related quality of life in pediatric patients with irritable bowel syndrome: a comparative analysis. J Dev Behav Pediatr 2006; 27(6): 451–458. 17164617 10.1097/00004703-200612000-00001

[43] Moore AR Eccleston C Derry S , et al. “Evidence” in chronic pain—establishing best practice in the reporting of systematic reviews. Pain 2010; 150(3): 386–389. 20627575 10.1016/j.pain.2010.05.011

[44] Saps M van Tilburg MAL Lavigne JV , et al. Recommendations for pharmacological clinical trials in children with irritable bowel syndrome: the Rome foundation pediatric subcommittee on clinical trials. Neurogastroenterol Motil 2016; 28(11): 1619–1631. 27477090 10.1111/nmo.12896

[45] Hicks CL von Baeyer CL Spafford PA , et al. The Faces Pain Scale-Revised: toward a common metric in pediatric pain measurement. Pain 2001; 93(2): 173–183. 11427329 10.1016/S0304-3959(01)00314-1

[46] Lalouni M Olén O Bjureberg J , et al. Validation of child-adapted short scales for measuring gastrointestinal-specific avoidance and anxiety. Acta Paediatr 2022; 111(8): 1621–1627. 35545865 10.1111/apa.16403 PMC9545055

[47] Trieschmann K Chang L Park S , et al. The visceral sensitivity index: a novel tool for measuring GI-symptom-specific anxiety in inflammatory bowel disease. Neurogastroenterol Motil 2022; 34(9): e14384. 10.1111/nmo.14384 PMC9427691 35478469

[48] von Gontard A Moritz A Thome-Granz S , et al. Abdominal pain symptoms are associated with anxiety and depression in young children. Acta Paediatr 2015; 104(11): 1156–1163. 26194632 10.1111/apa.13134

[49] Gulewitsch MD Weimer K Enck P , et al. Stress reactivity in childhood functional abdominal pain or irritable bowel syndrome. Eur J Pain 2017; 21(1): 166–177. 27470170 10.1002/ejp.914

[50] Borenstein M Hedges LV Higgings JPT , et al. Effect sizes based on means. In: Introduction to Meta-Analysis. Wiley; 2009. pp.21–32.

[51] Linardon J. Rates of attrition and engagement in randomized controlled trials of mindfulness apps: systematic review and meta-analysis. Behav Res Ther 2023; 170: 104421. 37862854 10.1016/j.brat.2023.104421

[52] Forbes A Keleher MR Venditto M , et al. Assessing patient adherence to and engagement with digital interventions for depression in clinical trials: systematic literature review. J Med Internet Res 2023; 25: e43727. 10.2196/43727 PMC10457707 37566447

[53] van Ballegooijen W Cuijpers P van Straten A , et al. Adherence to internet-based and face-to-face cognitive behavioural therapy for depression: a meta-analysis. PLoS One 2014; 9(7): e100674. 10.1371/journal.pone.0100674 PMC4100736 25029507

[54] Alfonsson S Olsson E Hursti T. Motivation and treatment credibility predicts dropout, treatment adherence, and clinical outcomes in an internet-based cognitive behavioral relaxation program: a randomized controlled trial. J Med Internet Res 2016; 18(3): e52. 10.2196/jmir.5352 PMC4804106 26957354

[55] Vasant DH Hasan SS Cruickshanks P , et al. Gut-focused hypnotherapy for children and adolescents with irritable bowel syndrome. Frontline Gastroenterol 2021; 12(7): 570–577. 34917314 10.1136/flgastro-2020-101679 PMC8640435

[56] Császár-Nagy N Bókkon I. Hypnotherapy and IBS: implicit, long-term stress memory in the ENS? Heliyon 2023; 9(1): e12751. 10.1016/j.heliyon.2022.e12751 PMC9849985 36685398

[57] Devenney J Hasan SS Morris J , et al. Clinical trial: predictive factors for response to gut-directed hypnotherapy for refractory irritable bowel syndrome, a post hoc analysis. Aliment Pharmacol Ther 2024; 59(2): 269–277. 37927144 10.1111/apt.17790

[58] Lövdahl J Blomqvist-Storm M Palsson OS , et al. Nurse-administered gut-directed hypnotherapy for irritable bowel syndrome: a two-year follow-up study. United European Gastroenterol J. Epub ahead of print June 2025. DOI: 10.1002/ueg2.70060. PMC12463711 40491242

