# Supplementary material for: Hypnotherapy self-exercises with audio files for children and adolescents with disorders of gut-brain interaction—a study of feasibility and preliminary effects in Sweden
Source: Ther Adv Gastroenterol. 2025 Sep 28;18:17562848251381141. doi: 10.1177/17562848251381141 (PMC12477360; doi:10.1177/17562848251381141)
Supplement: sj-docx-2-tag-10.1177_17562848251381141 – Supplemental material for Hypnotherapy self-exercises with audio files for children and adolescents with disorders of gut-brain interaction—a study of feasibility and preliminary effects in Sweden [file sj-docx-2-tag-10.1177_17562848251381141.docx]

Scales and questionnaires used in the study

| Measured parameter | Scale name | Abbreviation | Measured children (C) parents (P) | Timepoints  (weeks) |
| --- | --- | --- | --- | --- |
| Gastrointestinal symptoms | the Paediatric Quality of Life Inventory- gastrointestinal symptom short scale | Peds-QL gastro | C and P | 0,3,6,9,12 |
| Pain intensity | Faces Pain scale | Faces | C and P | 0,3,6,9,12 |
| Pain Frequency | Days of worst pain/week | Pain frequency | C and P | 0,3,6,9,12 |
| Quality of life | The Paediatric Quality of Life Inventory- quality of life | Peds-QL QOL | C and P | 0,3,6,9,12 |
| Avoidance behavior | Behavioral response questionnaire | BRQ-C | C | 0,3,6,9,12 |
| Anxiety related to GI-symptoms | Visceral sensitivity index | VSI-C | C | 0,3,6,9,12 |
| Stress | Pressure-activation stress scale | PAS-C | C | 0,3,6,9,12 |
| Depression | Child depression index | CDI-S | C | 0,12 |
| Anxiety | Spence Children’s Anxiety Scale | SCAS-C | C | 0,12 |
| School absence | Days/week |  | P | 0,12 |
| Credibility |  | c-scale | C and P | 3 |
| Treatment satisfaction | Client satisfaction questionnaire | CSQ-8 | C and P | 12 |
| Adequate relief | Subject’s global assessment of relief | SGA | C and P | 12 |
|  |  |  |  |  |
